# Lung perfusion scintigraphy to detect chronic lung allograft dysfunction after living-donor lobar lung transplantation

**DOI:** 10.1038/s41598-020-67433-4

**Published:** 2020-06-29

**Authors:** Haruchika Yamamoto, Seiichiro Sugimoto, Takeshi Kurosaki, Kentaroh Miyoshi, Shinji Otani, Mikio Okazaki, Masaomi Yamane, Takahiro Oto, Shinichi Toyooka

**Affiliations:** 1 Department of General Thoracic Surgery, Okayama University Hospital, 2-5-1 Shikata-cho, Kita-ku, Okayama-City, Okayama 700-8558 Japan; 2 Department of Organ Transplant Center, Okayama University Hospital, 2-5-1 Shikata-cho, Kita-ku, Okayama-City, Okayama 700-8558 Japan

**Keywords:** Respiratory tract diseases, Imaging, Diagnostic markers, Allotransplantation, Respiration

## Abstract

Because chronic lung allograft dysfunction (CLAD) develops predominantly on one side after bilateral living-donor lobar lung transplantation (LDLLT), lung perfusion scintigraphy (Q-scinti) was expected to show a perfusion shift to the contralateral unaffected lung with the development of CLAD. Our study examined the potential usefulness of Q-scinti in the diagnosis of CLAD after bilateral LDLLT. We conducted a single-center retrospective cohort study of 58 recipients of bilateral LDLLT. The unilateral shift values on Q-scinti were calculated and compared between the CLAD group (N = 27) and the non-CLAD group (N = 31) from 5 years before to 5 years after the diagnosis of CLAD. The unilateral shift values in Q-scinti were significantly higher in the CLAD group than in the non-CLAD group from 5 years before the diagnosis of CLAD to 5 years after the diagnosis (*P* < 0.05). The unilateral shift values in Q-scinti were significantly correlated with the percent baseline values of the forced expiratory volume in 1 s (*P* = 0.0037), the total lung capacity (*P* = 0.0028), and the forced vital capacity (*P* = 0.00024) at the diagnosis of CLAD. In patients developing unilateral CLAD after bilateral LDLLT, Q-scinti showed a unilateral perfusion shift to the contralateral unaffected lung. Thus, Q-scinti appears to have the potential to predict unilateral CLAD after bilateral LDLLT.

## Introduction

Recipients of lung transplantation (LT) still have a worse long-term survival than heart, liver, or kidney recipients^1–3^. The recipients of LT mainly succumb to chronic lung allograft dysfunction (CLAD)^1–3^ in the long term after both cadaveric LT and living-donor lobar lung transplantation (LDLLT)^4^. For the diagnosis of CLAD after LDLLT, lung ventilation scintigraphy was previously shown to be beneficial using ^133^Xe washout imaging^5^. However, because ^133^Xe was discontinued in 2016 and is no longer available in the world, a new diagnostic approach has been sought for CLAD after LDLLT.

Lung perfusion scintigraphy (Q-scinti) has been shown to be valuable for the diagnosis of CLAD after single LT, as follows^6, 7^. After single LT, Q-scinti normally shows a lung perfusion shift to the transplanted lung, rather than to the native lung, because of the lower vascular resistance of the graft; however, in patients developing CLAD after single LT, the perfusion decreases in the lung affected by CLAD, with a perfusion shift toward the native lung^6, 7^.

Interestingly, even in patients undergoing bilateral LDLLT, CLAD predominantly develops in a unilateral lung because in bilateral LDLLT, the lobar lung transplants are obtained from two different donors with different immunological backgrounds^4, 8^. Therefore, we considered that in patients developing CLAD after bilateral LDLLT, Q-scinti could show a decrease in the perfusion of the lung affected by CLAD and a perfusion shift toward the contralateral unaffected lung. Our study evaluated the usefulness of Q-scinti in the diagnosis of CLAD, which is predominantly unilateral, in patients who have undergone bilateral LDLLT.

## Results

**Figure 1:**
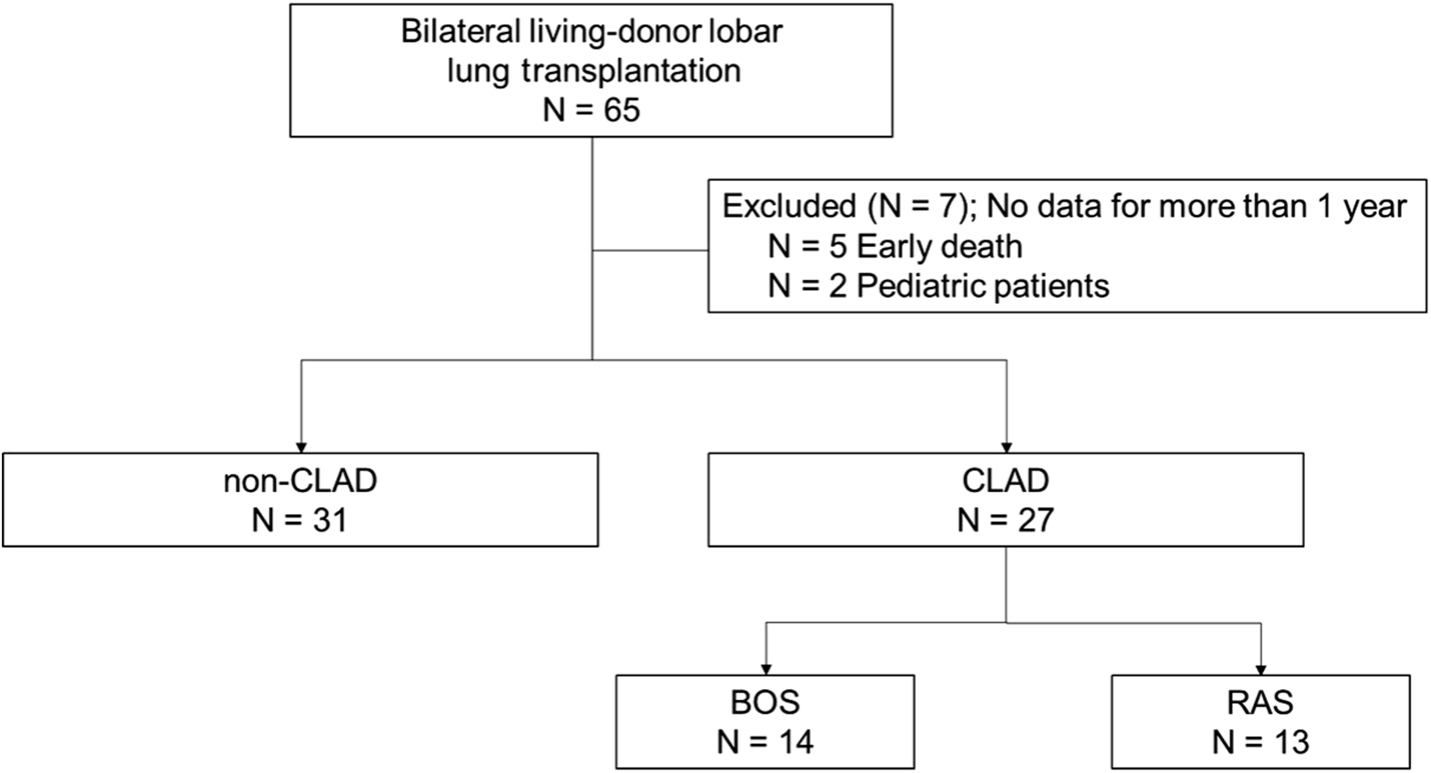
Flow-chart of the study cohort. Bilateral living-donor lobar lung transplantation (LDLLT) was performed in 65 patients during the study period. Seven patients, including five patients who died within 1 year of the LDLLT and two pediatric patients in whom lung perfusion scintigraphy (Q-scinti) could not be performed, were excluded from this study. Of the remaining 58 patients, 27 patients who developed chronic lung allograft dysfunction (CLAD) were designated as the CLAD group (N = 27), and 31 patients who did not develop CLAD were designated as the non-CLAD group (N = 31).

Table 1 shows the patient characteristics, and a schematic diagram of this study cohort is shown in Fig. 1. The risk factors for CLAD^9^ did not differ between the two groups, including human leukocyte antigen mismatches, cytomegalovirus mismatches, the incidence of primary graft dysfunction, the incidence of acute rejection, and the incidence of gastroesophageal reflux disease. The median observation period after bilateral LDLLT in the CLAD group (median, 3,733 days; range 679–6,298 days) was similar to that in the non-CLAD group (median, 3,907 days; range 722–5,931 days), and the median time to the onset of CLAD after LDLLT was 2,176 days (326–4,763 days) in the CLAD group.

**Table 1:** Patient characteristics.

	Non-CLAD (N = 31)	CLAD (N = 27)	*P* value
Preoperative variables			
Age (years), median (range)	32 (7–55)	31 (8–64)	0.69
Gender			
Male	6 (19.4%)	6 (22.2%)	0.78
Female	25 (80.6%)	21 (77.8%)	
Body mass index, median (range)	16.7 (10.8–26.6)	18.5 (10.5–29.0)	0.044
Diagnoses			
Interstitial lung disease	11 (35.5%)	9 (33.3%)	0.18
Pulmonary hypertension	9 (29.0%)	9 (33.3%)	
Pulmonary graft-versus-host disease	6 (19.4%)	1 (3.7%)	
Emphysema	0 (0%)	1 (3.7%)	
Bronchiectasis	0 (0%)	3 (11.1%)	
Other diseases	5 (16.1%)	4 (14.8%)	
Preoperative use of glucocorticoids, yes	15 (48.4%)	9 (33.3%)	0.29
Preoperative diabetes mellitus, yes	2 (6.5%)	1 (3.7%)	1
Lung allocation score, median (range)	46.0 (34.3–89.5)	40.8 (33.1–86.0)	0.068
CMV mismatch (recipient negative/donor positive), yes	6 (19.4%)	1 (3.7%)	0.11
Total number of HLA-A, HLA-B and HLA-DR mismatches, median (range)	5.5 (2–11)	7 (3–10)	0.078
Intraoperative variables			
Operative time (min), median (range)	444 (307–785)	477 (335–615)	0.74
Ischemic time (min), median (range)	163 (74–221)	169 (84–243)	0.29
Cardiopulmonary bypass use, yes	31 (100%)	27 (100%)	1
Postoperative variables			
Maximum grade of PGD (0–72 h), median (range)	1 (0–2)	0 (0–2)	0.50
Intravenous administration of calcineurin inhibitor, yes	9 (29.0%)	3 (11.1%)	0.12
Basiliximab usage, yes	8 (25.8%)	4 (14.8%)	0.35
Acute rejection, yes	17 (54.8%)	16 (59.3%)	0.80
Antibody-mediated rejection, yes	4 (12.9%)	1 (3.7%)	0.36
Postoperative GERD, yes	1 (3.2%)	1 (3.7%)	1
Time since transplant to follow-up (day), median (range)	3,907 (722–5,931)	3,733 (679–6,298)	0.96

Data are presented as n, median (range) or n (%).

*CMV* cytomegalovirus, *GERD* gastroesophageal reflux disease, *HLA* human leukocyte antigen, *PGD* primary graft dysfunction.

**Figure 2:**
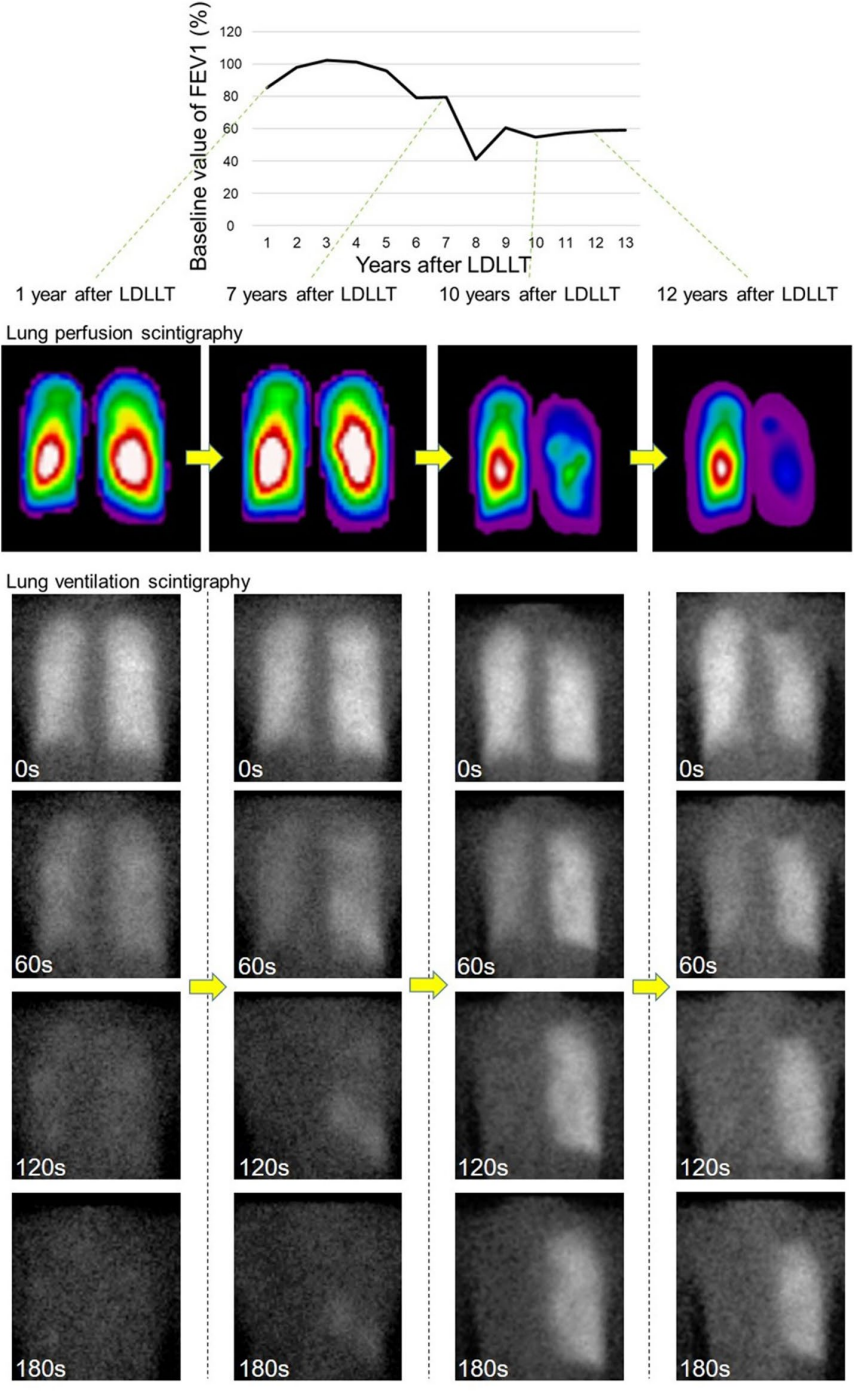
Representative course of unilateral chronic lung allograft dysfunction (CLAD) after bilateral living-donor lobar lung transplantation (LDLLT). Lung perfusion scintigraphy demonstrated a perfusion shift to the contralateral unaffected lung in the patient with unilateral CLAD after bilateral LDLLT, which was also associated with a decline in the forced expiratory volume in 1 s (FEV1) associated with CLAD. The lung ventilation scintigraphy showed that the washout of radionuclide tracer became slower, indicating air trapping in the lung affected with unilateral CLAD.

**Figure 3:**
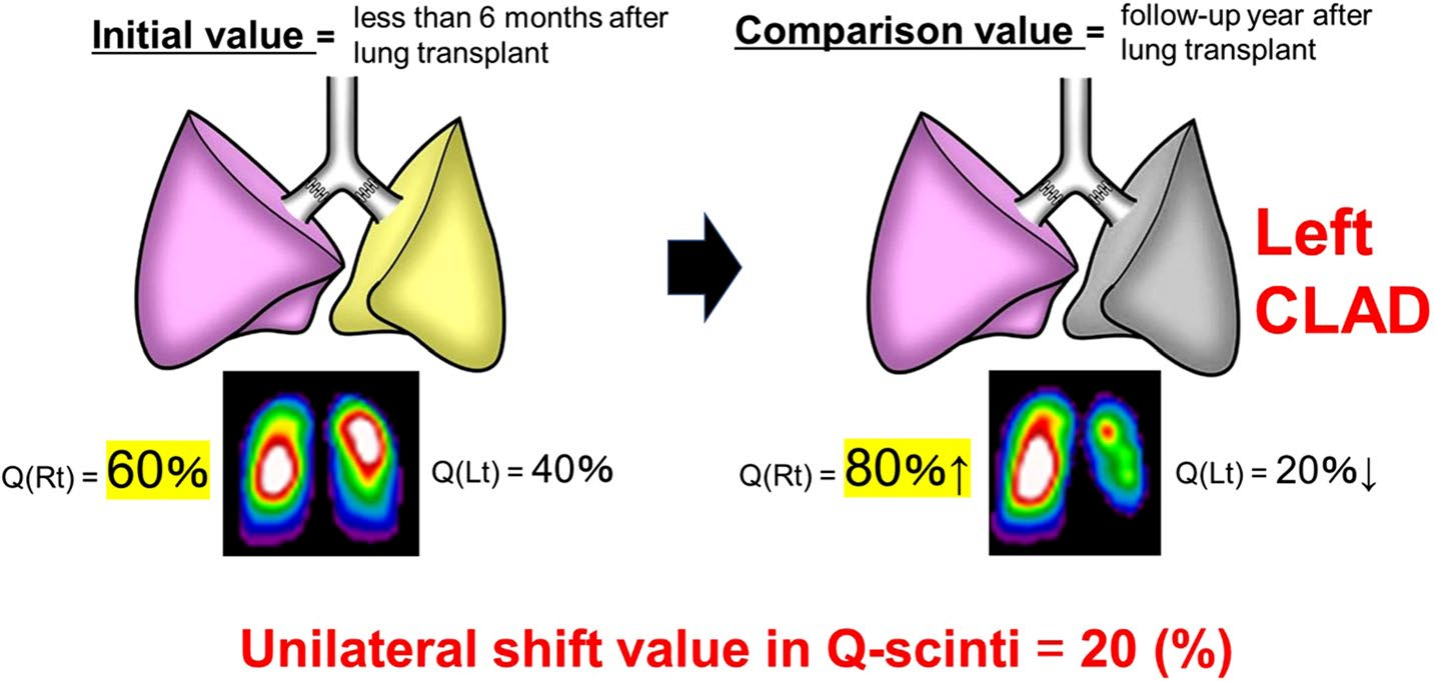
Formula for calculating the unilateral shift value in lung perfusion scintigraphy (Q-scinti). For example, the perfusion rate of the right lung was 60% at 3 months after a bilateral living-donor lobar lung transplantation (LDLLT), which was defined as the initial value. Then, the perfusion rate of the right lung increased to 80% because of chronic lung allograft dysfunction (CLAD) of the left lung at the follow-up conducted one year after bilateral LDLLT; this value was defined as the comparison value. For this case, the unilateral shift value in Q-scinti was calculated as 20% according to the equation. The lung images in the figure were created by Hidetoshi Yanase, who provided permission for their publication.

**Figure 4:**
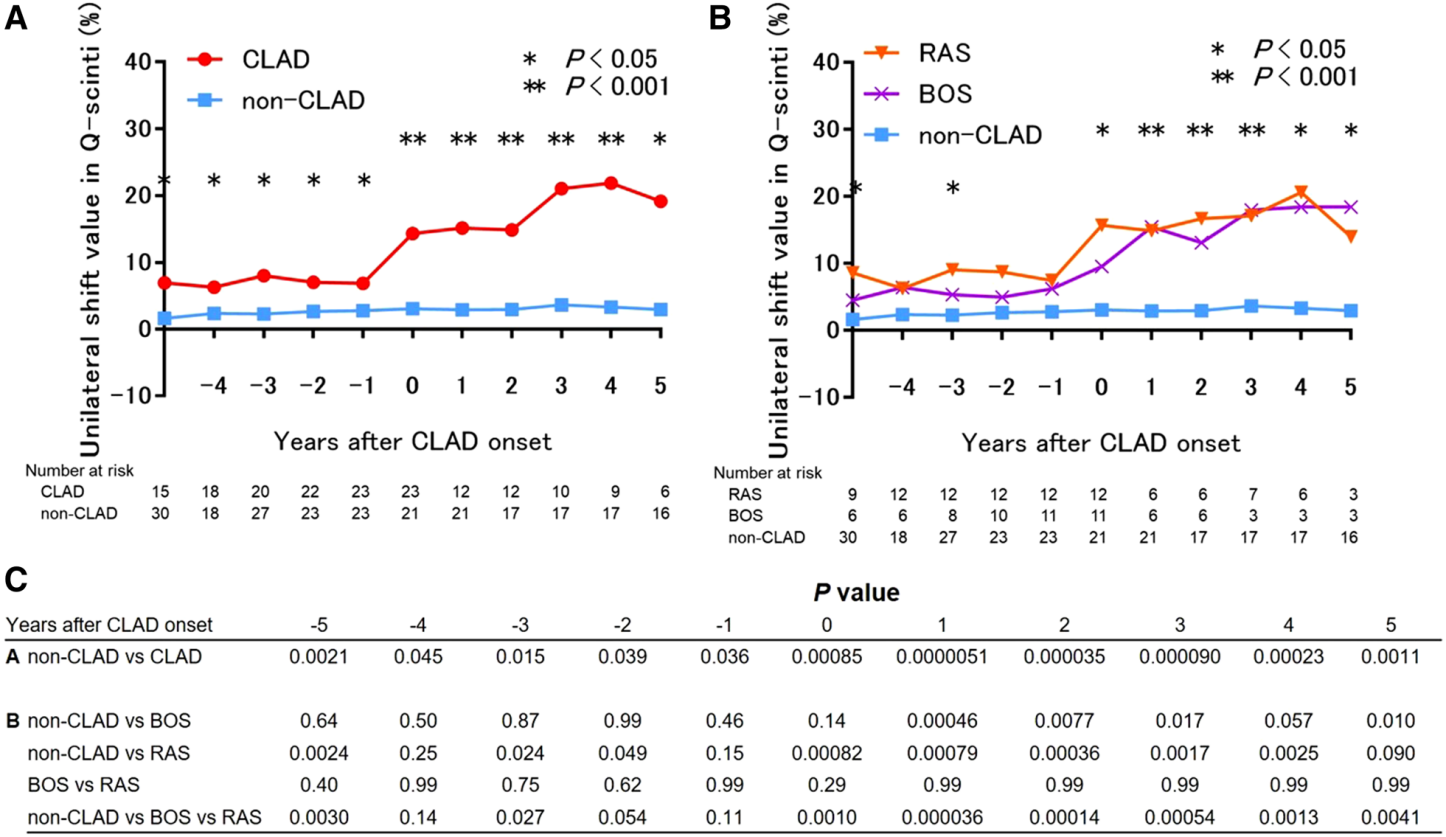
Postoperative changes in the unilateral shift values in lung perfusion scintigraphy (Q-scinti) after bilateral living-donor lobar lung transplantation (LDLLT). (**A**) The unilateral shift values in Q-scinti were significantly higher in the chronic lung allograft dysfunction (CLAD) group than in the non-CLAD group from 5 years before the diagnosis of CLAD to 5 years after the diagnosis of CLAD. (**B**) In the CLAD group, there was no significant difference in the unilateral shift value in Q-scinti between the group with bronchiolitis obliterans syndrome (BOS) and that with restrictive allograft syndrome (RAS), and the unilateral shift values in Q-scinti in both of the CLAD subgroups, BOS and RAS, were significantly higher than the value in the non-CLAD group from the time of CLAD diagnosis until 5 years after diagnosis. (**C**) The *P* value is for the comparison between groups in each year before and after the onset of CLAD.

**Figure 5:**
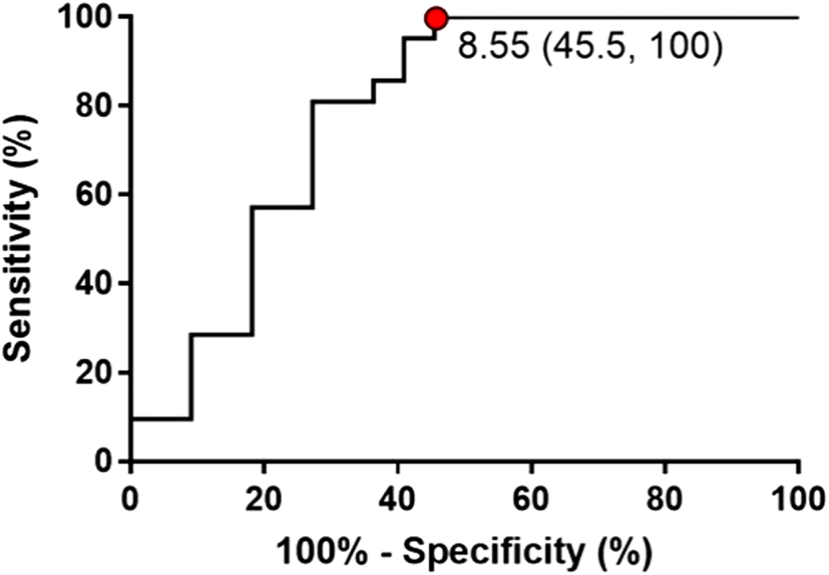
Receiver operating characteristic curve analysis to determine the performance of the unilateral shift value in lung perfusion scintigraphy for the diagnosis of chronic lung allograft dysfunction. The analysis yielded an area under the curve of 0.79, with a sensitivity of 100% and a specificity of 54% at a cutoff value of 8.55%.

**Figure 6:**
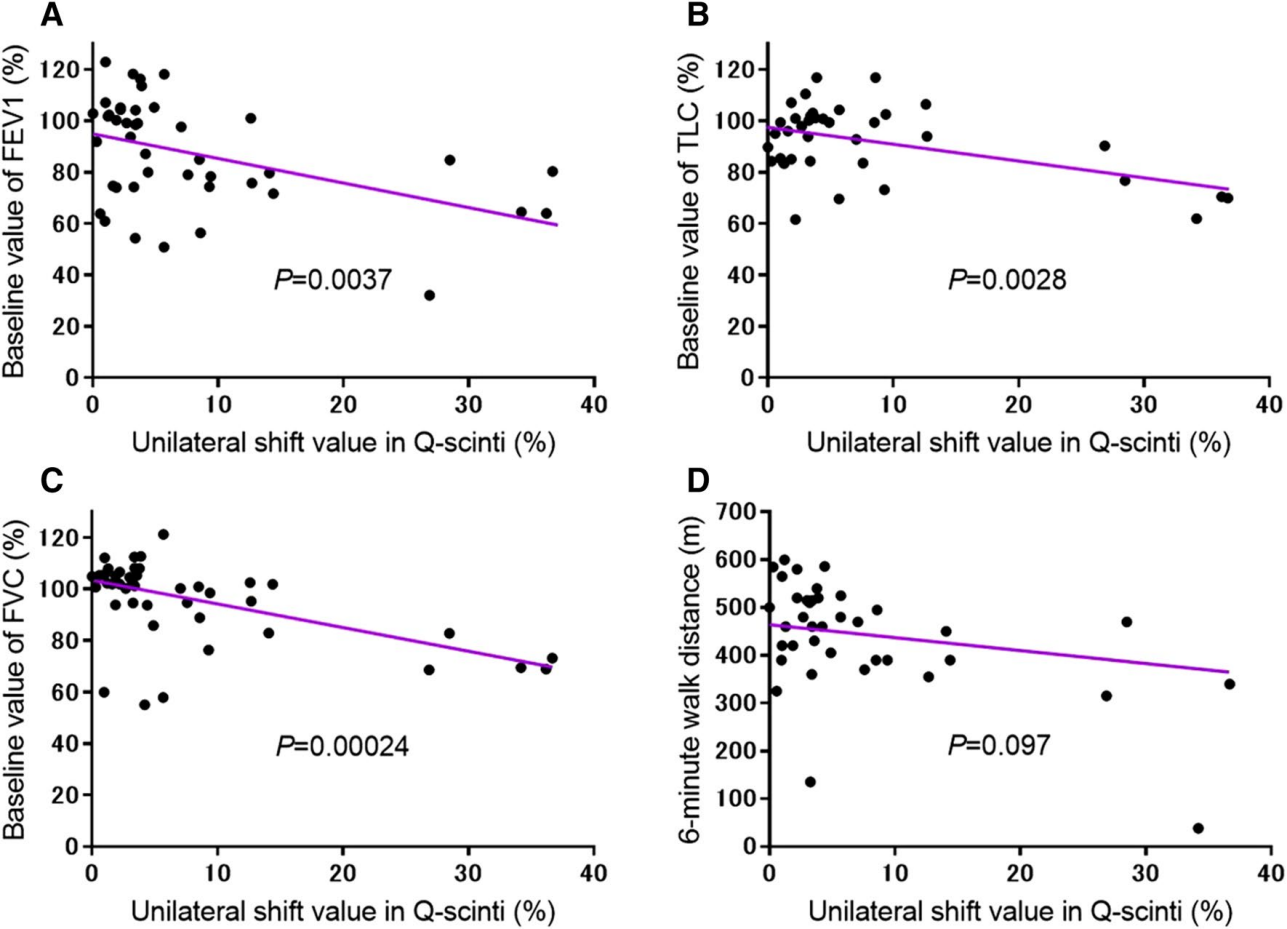
Correlation between the unilateral shift values in lung perfusion scintigraphy (Q-scinti) and (**A**) the percent baseline values of the forced expiratory volume in 1 s (FEV1), (**B**) the total lung capacity (TLC), (**C**) the forced vital capacity (FVC), and (**D**) the 6-min walk distance (6-MWD). There was a significant correlation between the unilateral shift values in Q-scinti and the percent baseline values of the FEV1, TLC and FVC, but not the 6-MWD, at the time of the diagnosis of chronic lung allograft dysfunction (CLAD) after bilateral living-donor lobar lung transplantation (LDLLT), as determined using the Pearson product-moment correlation coefficient (FEV1, *P* = 0.0037, r = − 0.43; TLC, *P* = 0.0028, r = − 0.47; FVC, *P* = 0.00024, r = − 0.53; 6-MWD, *P* = 0.097, r = − 0.27).

**Figure 7:**
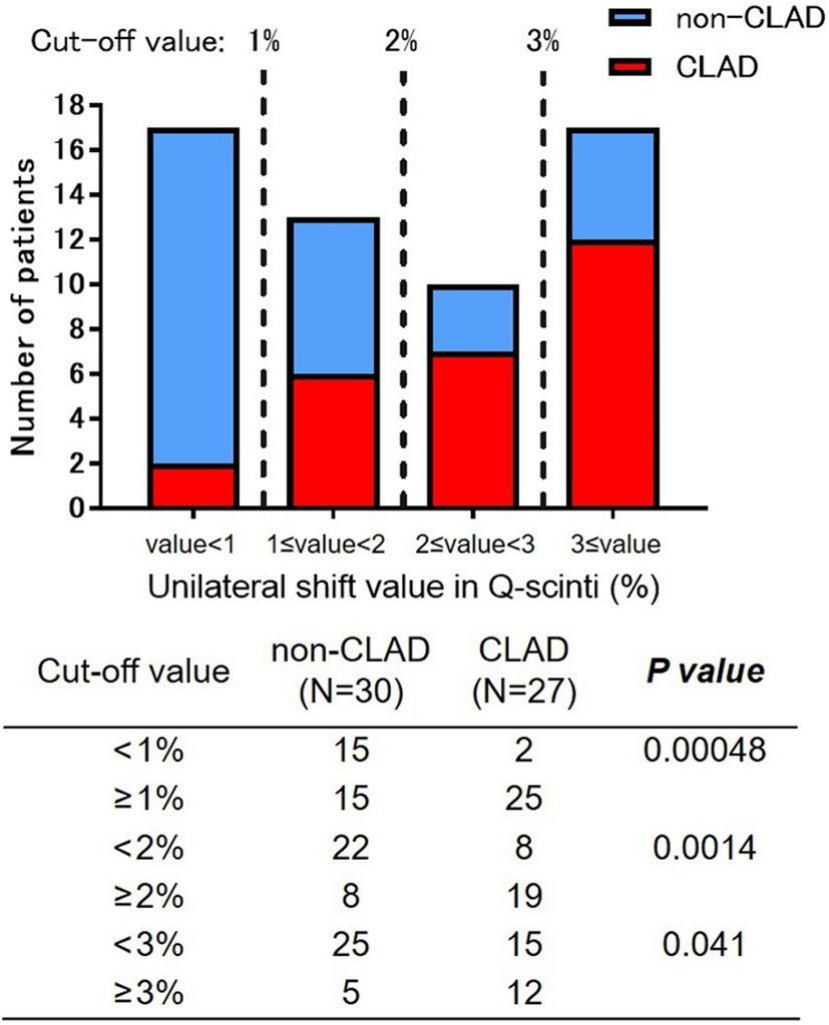
Prevalence of subsequent CLAD according to the unilateral shift values in lung perfusion scintigraphy (Q-scinti) at 1 year after bilateral living-donor lobar lung transplantation (LDLLT). The prevalence of subsequent CLAD was significantly higher in the patients with a unilateral shift value in Q-scinti ≥ 1% at 1 year after LDLLT than in those with a unilateral shift value in Q-scinti < 1% (*P* = 0.00048). Applying a cut-off value of 2% or 3% of the unilateral shift value in Q-scinti also resulted in significant differences in the prevalence of subsequent CLAD.

As shown in Fig. 2, in the recipients with unilateral CLAD after bilateral LDLLT, Q-scinti demonstrated a perfusion shift towards the contralateral unaffected lung. Unsurprisingly, the percent baseline value of the forced expiratory volume in 1 s (FEV1) in the CLAD group was significantly lower than that in the non-CLAD group at and after the diagnosis of CLAD. Notably, the unilateral shift values in Q-scinti, as shown in Fig. 3, were significantly higher in the CLAD group than in the non-CLAD group from even 5 years before the diagnosis of CLAD to 5 years after the diagnosis of CLAD (Fig. 4A). Furthermore, in the CLAD group, there was no significant difference in the unilateral shift value in Q-scinti between the group with bronchiolitis obliterans syndrome (BOS) and restrictive allograft syndrome (RAS), and the unilateral shift values in Q-scinti in both the CLAD subgroups with BOS and RAS were significantly higher than the value in the non-CLAD group from the time of the diagnosis of CLAD to 5 years after the diagnosis (Fig. 4B). A receiver operating characteristic curve (ROC) analysis to determine the performance of the unilateral shift value in Q-scinti for the diagnosis of CLAD yielded an area under the curve of 0.79, with a sensitivity of 100% and a specificity of 54% at a cutoff value of 8.55% (Fig. 5). Furthermore, at the diagnosis of CLAD after bilateral LDLLT, the unilateral shift values in Q-scinti were moderately but significantly correlated with the percent baseline values of FEV1, the total lung capacity (TLC), and the forced vital capacity (FVC) (FEV1, *P* = 0.0037, r = − 0.43; TLC, *P* = 0.0028, r = − 0.47; FVC, *P* = 0.00024, r = − 0.53), but not the 6-min walk distance (*P* = 0.097, r = − 0.27) (Fig. 6). The prevalence of subsequent CLAD was significantly higher in the patients with a unilateral shift value in Q-scinti ≥ 1% at 1 year after LDLLT than in those with a unilateral shift value in Q-scinti < 1% (Fig. 7).

## Discussion

In this study, after bilateral LDLLT, the unilateral shift value in Q-scinti was significantly higher in patients with CLAD than in patients without CLAD from 5 before to 5 years after the diagnosis of CLAD. Moreover, the unilateral shift values in Q-scinti did not differ across subgroups (BOS and RAS) throughout the study period, and both the BOS and RAS subgroups showed significantly increased unilateral shift values in Q-scinti at CLAD diagnosis, compared with those in the non-CLAD patients. Furthermore, the unilateral shift values in Q-scinti were significantly correlated with the percent baseline values of FEV1, TLC and FVC at the diagnosis of CLAD. Our results suggest that the unilateral perfusion shift to the unaffected lung in Q-scinti could predict the development of unilateral CLAD after bilateral LDLLT. To the best of our knowledge, this is the first report to focus on Q-scinti as a potential diagnostic tool for CLAD after bilateral LDLLT.

CLAD after bilateral LDLLT develops unilaterally^4, 8^ because the transplanted lung lobes in bilateral LDLLT originate from two different donors with different immunological backgrounds. The diagnosis of CLAD is based on pulmonary function tests^10, 11^. However, after bilateral LDLLT, the unaffected contralateral lung might mask the functional decline and delay the diagnosis of CLAD. Until 2016, ventilation scintigraphy with ^133^Xe washout imaging was used for the diagnosis of unilateral CLAD after bilateral LDLLT^5^, but the use of ^133^Xe was discontinued. As an alternative diagnostic tool, inspiratory and expiratory computed tomography volumetry has been used to detect unilateral CLAD after bilateral LDLLT, but this volumetric method rarely allows the early detection of CLAD^12^. By contrast, our study showed that the perfusion shift in Q-scinti predicts the development of unilateral CLAD from 5 years before the diagnosis of CLAD after bilateral LDLLT. Moreover, the perfusion shift in Q-scinti was correlated with the postoperative changes in FEV1, TLC and FVC at the onset of CLAD. Even in patients with low perfusion shift percentages at 1 year after LDLLT, the prevalence of subsequent CLAD was significantly higher than in those without a perfusion shift. Therefore, Q-scinti appears to have excellent potential for the early screening of patients at risk for the development of unilateral CLAD after bilateral LDLLT. As LDLLT is currently performed exclusively in Japan, this diagnostic approach might be useful only in this country, but this approach could potentially lead to further examination and enhanced immunosuppression for the early diagnosis and prompt treatment of unilateral CLAD in these patients.

Theoretically, the distribution of the lung perfusion after bilateral LDLLT depends on the lung volume of the transplanted lung lobes, since the lung grafts from healthy living donors have normal vascular resistance in intact vascular beds. When the graft function deteriorates after bilateral LDLLT, the lung perfusion shifts toward the contralateral unaffected lung because of the relatively lower vascular resistance in that lung. In this study, a cutoff value of 8.55% for unilateral shift was found to have a sensitivity of 100% and a specificity of 54% for the diagnosis of CLAD at the time of CLAD diagnosis after bilateral LDLLT. Furthermore, the lung perfusion shift to the unaffected lung in Q-scinti became more significant when the FEV1 started declining in patients with CLAD. These findings indicate that in patients developing CLAD after bilateral LDLLT, the decrease in perfusion of the affected lung might precede the changes in airway compliance, as described for single LT^7^. This result is consistent with former animal experiments, demonstrating that a decrease in lung perfusion leads to a decrease in pulmonary function^13^. The decrease in the lung perfusion in the affected lung with CLAD might be attributable to the inflammatory process of CLAD or graft vasculopathy^14^. Further study is required to elucidate the mechanisms of this decrease in lung perfusion and how it could lead to a decline in FEV1 in patients developing CLAD after bilateral LDLLT.

Regarding radiation exposure, the dose during Q-scinti was 1.48–3.00 mGy/37 MBq, and the required dose was relatively low, at 0.8 mSV; in contrast, the radiation dose for chest X-ray is 0.2 mSV and that for contrast-enhanced computed tomography ranges from 1.6 to 8.3 mSV^6, 7^. In general, Q-scinti is used for the diagnosis of pulmonary thrombosis, even in pregnant women with acute pulmonary thrombosis^6, 7^. Given these factors, Q-scinti is considered to be a potentially acceptable diagnostic tool for the long-term follow-up of patients after bilateral LDLLT.

The present study had several limitations. First, this was a retrospective study conducted at a single transplant institution. Second, the number of LDLLT recipients and CLAD patients was relatively small in our study. Third, in some cases, the follow-up period was of intermediate duration, and longer follow-up periods are needed to validate the prognostic impact of CLAD, although the follow-up period was more than one year in all the patients. Fourth, because we set the unilateral shift values in Q-scinti at 6 years after LDLLT as the control value in the non-CLAD group, the time point of the unilateral shift values in Q-scinti differed between the CLAD and non-CLAD groups. However, given the fact that LDLLT is currently performed exclusively in Japan, our study provides practical information for the long-term follow-up and diagnosis of CLAD after bilateral LDLLT.

In conclusion, after bilateral LDLLT, the unilateral perfusion shift to the contralateral, unaffected lung in Q-scinti was associated with the development of CLAD and increased after the onset of CLAD. Moreover, the lung perfusion shift in Q-scinti was associated with declines in the FEV1, TLC and FVC at the time of CLAD diagnosis after bilateral LDLLT. Based on our findings, we concluded that Q-scinti had the potential to predict unilateral CLAD after bilateral LDLLT.

## Methods

### Patients

This was a single-center retrospective cohort study in which 65 patients who had undergone bilateral LDLLT at Okayama University Hospital between October 1998 and October 2014 were initially enrolled. Seven patients, including two pediatric patients in whom Q-scinti could not be performed and five patients who died within 1 year of the LDLLT, were excluded from this study. No organs were procured from prisoners in this study. Of the remaining 58 patients, 27 patients who developed CLAD were designated as the CLAD group (N = 27), and 31 patients who did not develop CLAD were designated as the non-CLAD group (N = 31). The CLAD group was divided into the BOS group (N = 14) and the RAS group (N = 13) according to CLAD phenotype.

The triple immunosuppressive therapy consisted of calcineurin inhibitors including tacrolimus or cyclosporine, mycophenolate mofetil or azathioprine, and glucocorticoid after LDLLT. Calcineurin inhibitor was enterically administered through a nasogastric tube for patients treated between 1998 and 2010 or was intravenously administered for patients treated between 2011 and 2014, followed by oral administration early after LDLLT^15^. Basiliximab was administered to patients at risk of developing renal dysfunction.

Pulmonary function testing and Q-scinti were performed at 3, 6 and 12 months after LT and every year thereafter. Until 2016, lung ventilation scintigraphy was performed at the same time as Q-scinti to diagnose CLAD using ^133^Xe washout imaging^5^. According to the classification system proposed by the International Society of Heart and Lung Transplantation (ISHLT)^10, 11^, obstructive CLAD or BOS was defined as an irreversible decline of the FEV1 to < 80% of the baseline value, and restrictive CLAD or RAS was defined as an irreversible decline of both the FEV1 to < 80% and the total lung capacity (TLC) to < 90% of the values measured at baseline^10, 11^. The baseline values of the FEV1 and TLC were calculated as the average of the two best values obtained at least 3 weeks apart.

Q-scinti was performed after the intravenous administration of 185 MBq of 99c-macroaggregated albumin (= ^99m^Tc-MAA, Techne MAA kit, FUJIFILM, JAPAN) with the patient in a supine position. Images in the anterior and posterior oblique views were obtained by collecting 7,000,000 counts per view on a single-head gamma camera (GCA901A/HG; Toshiba Medical) using a single photon emission computed tomography system (Symbia T16; Siemens Medical Solutions, USA) equipped with a parallel-hole collimator. Images obtained in the posterior view were used to calculate the relative perfusion.

For the differential diagnosis of CLAD, a blood examination, plain chest X-ray, computed tomography of the chest, 6-min walk test, electrocardiography, and echocardiography were also performed at the same time as the pulmonary function testing and Q-scinti. Transbronchial biopsy was not performed to diagnose CLAD after LDLLT to avoid unexpected bleeding or pneumothorax from the small lobar grafts^16^. The study protocol (No. 1901-026) was approved, and individual written informed consent was waived by the institutional review board of Okayama University Hospital. All the methods were performed in accordance with the relevant guidelines and regulations.

### Evaluation of lung perfusion scintigraphy

The unilateral shift values of lung perfusion in Q-scinti were calculated using the following equation (Fig. 3):

$${\mathbf{Unilateral}}\;{\mathbf{shift}}\;{\mathbf{value}} = \left| {{\mathbf{Initial}}\;{\mathbf{value}}\left( \% \right) - {\mathbf{Comparison}}\;{\mathbf{value}}\left( \% \right)} \right|,$$

where **“Initial value”** is the perfusion rate of the right lung (%) in Q-scinti within 6 months after bilateral LDLLT, and **“Comparison value”** perfusion rate of the right lung (%) in Q-scinti at the follow-up conducted one year after bilateral LDLLT.

The unilateral shift values in Q-scinti were compared between the CLAD group and the non-CLAD group from 5 before to 5 years after the diagnosis of CLAD. In the CLAD group, the median time to the onset of CLAD after bilateral LDLLT was 2,176 days (5.96 years), or nearly 6 years after LDLLT; thus, the unilateral shift value in Q-scinti at 6 years after bilateral LDLLT was designated as the control value in the non-CLAD group. In addition, in the CLAD group, a subgroup analysis was performed to compare the unilateral shift values in Q-scinti between the patients with BOS and those with RAS. To assess the potential for the early screening of CLAD, the prevalence of subsequent CLAD was examined according to the unilateral shift values in Q-scinti at 1 year after LDLLT.

### Statistical analysis

All the statistical analyses were performed using the GraphPad Prism 7.04 software (San Diego, CA, USA). The Student *t*-test was used to compare the changes in the unilateral shift values in Q-scinti between the CLAD and non-CLAD groups. The Bonferroni correction was used for multiple comparisons between two groups among the non-CLAD, BOS and RAS groups, and a one-way ANOVA was used for a three-factor comparison analysis among the non-CLAD, BOS and RAS groups. Missing data were not replaced. Differences in the patient characteristics between two groups were tested using the Mann–Whitney U test for continuous variables and the Pearson chi-square test for categorical variables. Differences were considered significant at *P* < 0.05. An ROC analysis was performed to determine the optimal cutoff value for the unilateral shift value in Q-scinti between two groups. The Pearson product-moment correlation coefficient was calculated between the unilateral shift values in Q-scinti and the percent baseline values of FEV1, TLC, FVC, or the 6-min walk distance at the diagnosis of CLAD after bilateral LDLLT.

## Data Availability

The datasets generated during and/or analyzed during the current study are available from the corresponding author upon reasonable request.
